# Enhancing Motor Network Activity Using Real-Time Functional MRI Neurofeedback of Left Premotor Cortex

**DOI:** 10.3389/fnbeh.2015.00341

**Published:** 2015-12-24

**Authors:** Theo F. Marins, Erika C. Rodrigues, Annerose Engel, Sebastian Hoefle, Rodrigo Basílio, Roberto Lent, Jorge Moll, Fernanda Tovar-Moll

**Affiliations:** ^1^D’Or Institute for Research and EducationRio de Janeiro, Brazil; ^2^Institute of Biomedical Sciences, Federal University of Rio de JaneiroRio de Janeiro, Brazil; ^3^Augusto Motta University (Unisuam)Rio de Janeiro, Brazil; ^4^Clinic for Cognitive Neurology, University Hospital LeipzigLeipzig, Germany; ^5^National Institute for Translational Neuroscience (INNT)Rio de Janeiro, Brazil

**Keywords:** neurofeedback, motor imagery, left premotor cortex, neuromodulation, motor rehabilitation

## Abstract

Neurofeedback by functional magnetic resonance imaging (fMRI) is a technique of potential therapeutic relevance that allows individuals to be aware of their own neurophysiological responses and to voluntarily modulate the activity of specific brain regions, such as the premotor cortex (PMC), important for motor recovery after brain injury. We investigated (i) whether healthy human volunteers are able to up-regulate the activity of the left PMC during a right hand finger tapping motor imagery (MI) task while receiving continuous fMRI-neurofeedback, and (ii) whether successful modulation of brain activity influenced non-targeted motor control regions. During the MI task, participants of the neurofeedback group (NFB) received ongoing visual feedback representing the level of fMRI responses within their left PMC. Control (CTL) group participants were shown similar visual stimuli, but these were non-contingent on brain activity. Both groups showed equivalent levels of behavioral ratings on arousal and MI, before and during the fMRI protocol. In the NFB, but not in CLT group, brain activation during the last run compared to the first run revealed increased activation in the left PMC. In addition, the NFB group showed increased activation in motor control regions extending beyond the left PMC target area, including the supplementary motor area, basal ganglia and cerebellum. Moreover, in the last run, the NFB group showed stronger activation in the left PMC/inferior frontal gyrus when compared to the CTL group. Our results indicate that modulation of PMC and associated motor control areas can be achieved during a single neurofeedback-fMRI session. These results contribute to a better understanding of the underlying mechanisms of MI-based neurofeedback training, with direct implications for rehabilitation strategies in severe brain disorders, such as stroke.

## Introduction

Neurofeedback (NFB) is a technique that allows individuals to voluntarily modulate their own neurophysiological responses via a feedback loop. Electroencephalography (EEG)-based NFB has been employed since the 1960s, and several studies have indicated that with appropriate training, healthy individuals and patients can learn to control their own brain activation, with a wide range of applications in neurological and psychiatric disorders (Birbaumer and Cohen, 2007). Some important limitations of surface EEG-based NFB are its poor spatial resolution and insensitivity for detecting subcortical brain activity. On the other hand, the fast and steady development of real time functional magnetic resonance imaging (fMRI) over the last decade, along with optimization of acquisition techniques and image processing algorithms (Cox et al., 1995) has opened up new perspectives for NFB research. One of the first published works using fMRI to study NFB (Yoo and Jolesz, 2002) demonstrated that healthy volunteers were able to increase the activation of cortical sensorimotor areas while performing a simple finger tapping task. Since this seminal study, mounting evidence has established that healthy subjects and patients can gain control over activation of different specific brain areas, such as the amygdala (Posse et al., 2003), anterior cingulate cortex (deCharms et al., 2005), and insular cortex (Caria et al., 2007), when receiving feedback about the activity in these regions.

In the field of motor learning and rehabilitation, motor imagery (MI) – defined as a dynamic state during which a subject mentally simulates a given action (for a review see Sharma et al., 2006) – has been established effective in recruiting the motor control network and in promoting motor skill learning and motor rehabilitation. Previous studies demonstrated that both motor execution and MI activate a common brain circuitry, including the supplementary motor area (SMA), premotor cortex (PMC), basal ganglia (BG), parietal areas, and anterior cerebellum (lobule VI) (Stephan et al., 1995; Lotze et al., 1999; Gerardin et al., 2000; Hanakawa et al., 2003). These regions were also shown to be involved in motor sequence learning (Tanji, 2001; Doyon and Benali, 2005; Matsuzaka et al., 2007). Further, a number of studies suggest that motor skill learning induced by MI elicits activation changes in the primary motor area (M1), SMA, cerebellum, parietal, and the visual cortices (Nyberg et al., 2006; Olsson et al., 2008; Ma et al., 2010; Zhang et al., 2011, 2012), regions that contribute to different aspects of motor skill learning and visuomotor integration.

Importantly, sensory and motor-targeted NFB approaches hold great potential for clinical application in motor rehabilitation (Sharma et al., 2006; Cramer et al., 2011). The sensorimotor network has been recently explored by fMRI NFB, using either MI or movement execution. A previous NFB study reported that healthy individuals, compared to a sham group (that unwittingly received false feedback), increased their activation in a region of interest (ROI) localized in the primary sensorimotor areas during MI (deCharms et al., 2004). However, another study reported up-regulation of M1 during motor exection but failed to show successful upmodulation in M1 (Berman et al., 2012) during MI tasks. In addition, it has also been shown that increased activation achieved during NFB training can be sustained (using a so-called transfer session) for few minutes or even weeks (Yoo et al., 2008) after NFB training. Despite the lack of upmodulation effects in the target ROI, previous studies have shown that NFB training during MI induced functional connectivity changes among motor brain areas (Hui et al., 2014) and showed that ROI activity was correlated with motor performance (Blefari et al., 2015).

Still, while most NFB studies on motor control have focused on the primary sensorimotor cortex, there is growing evidence in humans and other animals suggesting that plastic changes involving additional motor control regions occur in response to brain damage, leading to extensive functional and structural reorganization (Fridman et al., 2004; Dancause et al., 2005; Zeiler and Krakauer, 2013). In stroke patients, for example, the recruitment of spared non-primary motor-related areas, either ipsilesional or contralesional, may contribute to motor recovery (Ward, 2004). Particularly, a spared PMC appears to be important for motor recovery (Liu and Rouiller, 1999; Miyai et al., 1999; Fridman et al., 2004). It has been also suggested that the PMC may play an “executive” motor function, in patients with M1 lesions (Ward et al., 2003). In this context, remodeling and training the ipsilesional PMC may have a substantial impact in motor recovery. Nevertheless, although promising, only few studies have explored NFB-induced modulation in premotor areas and to our knowledge, so far no fMRI NFB controlled study has been successfully conducted to explore the effects of voluntary control of PMC activation using MI. Another important open question is whether the fMRI NFB-based modulation of PMC would extend to a wider motor control network, given its putative implication in rehabilitation strategies.

We here investigated (1) whether healthy human volunteers are able to increase the activation of left PMC during a finger tapping MI task while receiving real-time NFB information and (2) whether a successful modulation exerted influence over the motor control areas, beyond the PMC. In contrast to previous studies (deCharms et al., 2004; Yoo et al., 2008), we compared the NFB training to a CTL group, which received no feedback information, but watched equivalent but random visual stimuli, while being aware of their random nature. This avoided the frustration effect of not being able to control the feedback stimuli during the performance of MI by CTL participants.

## Materials and Methods

### Participants

Twenty-eight healthy volunteers were enroled in and completed the study. Half of the participants were assigned to the NFB group (9 female; mean age: 27.1 years, *SD* = 4.7 years, range 20–34 years) and the other half to the CTL group (9 female; mean age: 27.2 years, *SD* = 6.4 years, range 18–38 years). There were no age or sex differences between groups (*p* = 1; *t*-test). All participants were right-handed according to the Edinburgh handedness inventory (Oldfield, 1971), had no history of psychiatric or neurologic disease, and were not taking brain active medication. All participants gave their written informed consent to participate in the study. The experiment was performed in accordance with the ethical standards compliant with the Declaration of Helsinki and has been approved by the D’Or Institute Ethics and Scientific Committee.

### Experimental Procedure Before fMRI

**FIGURE 1:**
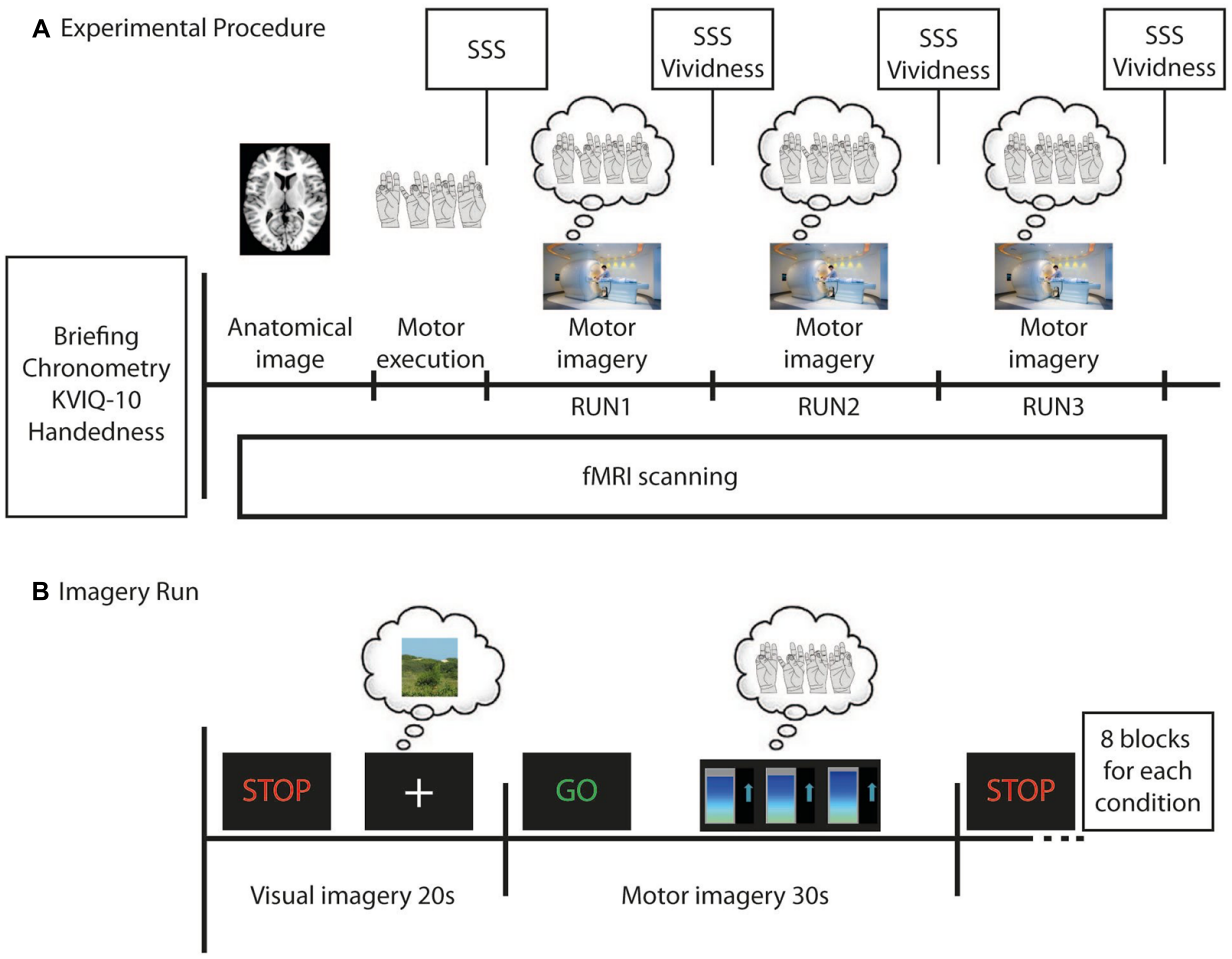
**Time course of the whole experiment **(A)** and of each imagery run **(B)** for each participant in the neurofeedback group (NFB) and control (CTL) group. (A)** Before functional magnetic resonance imaging (fMRI) scanning each participant was briefed about the experiment, filled out the Edinburgh Handedness Questionnaire, did the Kinesthetic and Visual Imagery Questionnaire (KVIQ-10), was asked to perform the finger tapping task and the motor imagery (MI) of the finger tapping (three times each) in order to calculate the mental chronometry. During fMRI scanning an anatomical image was first acquired. Next, participants performed the finger tapping task in the motor execution run, followed by three imagery runs. After each run, participants evaluated their sleepiness (using SSS) and, after each imagery run, the vividness of their MI. **(B)** During imagery runs participants were required to imagine a still landscape (for 20 s) after they have seen the STOP signal, and then imagine themselves doing finger tapping (for 30 s) after the GO signal. During MI all participants observed changing bar graphs that were either related to participant’s brain activity in the left premotor cortex (NFB group) or random (CTL group). Eight visual and eight MI blocks were alternated in each imagery run.

All participants underwent the same procedures before the imaging acquisition (**Figure 1A**), which consisted of briefing and behavioral training, and behavioral testing. *Briefing* comprised a standard explanation of the procedure during scanning and computer-based simulation of the task to be performed during fMRI scanning (practicing of motor execution and imagery of right hand finger tapping). *Behavioral training* included the presentation of the scanner noise and graphs that would be related to the brain activity during fMRI scanning, as well as right hand finger tapping with the index, middle, ring and little fingers on the thumb sequentially and repeatedly. The NFB group participants were instructed to try to increase their brain activity in the left PMC during MI, with the aid of a feedback display of the blood oxygen level dependent (BOLD) signal averaged within the left PMC. CTL group participants had to perform MI with their eyes open while watching random graphs displayed and were informed that these graphs should be watched for experimental reasons, but had no meaning. *Behavioral testing* comprised the Kinesthetic and Visual Imagery Questionnaire (KVIQ-10; Malouin et al., 2007), and chronometry measures. KVIQ-10 required participants first to perform five different movements once (overt execution of flexing the shoulder, thumb-fingers opposition, forward trunk flexion, hip abduction, foot tapping). After every single performance they had to imagine the same movement in a (first person) visual and a kinesthetic imagery condition each followed by a self-evaluation of the intensity on a 5-point scale (ranging from 5=as clear as seeing/as intense as execution, to 1=no image/no sensation to the visual and kinesthetic imagery, respectively). The final scores in **Table 1A** represent the mean scores from the five MI self-evaluations (visual and kinesthetic). Furthermore, chronometry measures were verbally collected for three finger tapping sequences in (i) overt motor execution and (ii) kinesthetic MI. Mental chronometry scores were calculated as a ratio between the difference of time for execution of three complete sequences of finger tapping movements and the time for MI. This difference was divided by the time for MI and multiplied by 100 in order to obtain percent values (time measures were represented by the means over the three trials). Chronometry measures were obtained for 13 out of 14 participants in the NFB group and for all participants in the CTL group (**Table 1**). Because motor execution of a task and MI of the same task share common brain regions (Stephan et al., 1995; Lotze et al., 1999; Gerardin et al., 2000; Hanakawa et al., 2003), the time necessary to perform both tasks (motor execution and MI) should match, as the time taken to recruit those common network is assumed to be similar (Decety and Jeannerod, 1996). Thus, similar times in the chronometry measures (motor execution in comparison to MI) would indicate that the MI task was performed adequately.

**Table 1:** Behavioral measures for the neurofeedback group (NFB) and the control (CTL) group before (A) and during (B) functional magnetic resonance imaging (fMRI) scanning^∗^.

	NFB	CTL
**(A) Before fMRI scanning**		
Kinesthetic and Visual Imagery Questionnaire (KVIQ-10)		
Visual Imagery	4.6 ± 0.5	4.3 ± 0.9
Kinesthetic Imagery	3.7 ± 0.9	3.7 ± 0.9
Mental chronometry score		
Ratio of time: (imagery-execution)/imagery	24.0 ± 12.3	33.0 ± 33.7
**(B) During fMRI scanning**		
Standford Sleepiness Scale (SSS)		
RUN1	2.2 ± 1.3	2.3 ± 1.4
RUN3	2.5 ± 1.4	2.8 1.5
Vividness		
RUN1	3.1 ± 1.1	3.7 ± 1.1
RUN3	2.8 ± 1.1	3.6 ± 1.2
Accelerometer		
RUN1	1.21 ± 0.23	1.00 ± 0.21
RUN3	1.05 ± 0.26	1.01 ± 0.11

*^∗^Values indicate mean ± standard deviation for the NFB and the CTL group. RUN1, first run; RUN3, last run. KVIQ-10, Kinesthetic and Visual Imagery Questionnaire: mean for 5 actions rated on a scale from 5 (as intense as executing/seeing) – 1 (no sensation). Mental chronometry scores are in percent. Stanford Sleepiness Scale: 1 (active) – 7 (asleep); Vividness: 5 (as intense as executing) – 1 (no sensation). Accelerometer: values comprise the ratio motor/visual imagery of the RMS over time for the 3-dimensional thumb acceleration values in *g**.

### Experimental Procedure During fMRI

All participants were placed in a comfortable supine position inside the MRI scanner. A LCD display mounted in the scanner room was seen by the participants by way of a mirror system attached to the head coil, and used for stimulus delivery. Participants were instructed to keep their eyes open during the entire experiment. The fMRI task paradigm (**Figure 1A**) comprised one motor execution run (without NFB information) followed by three MI runs (RUN1, RUN2, and RUN3). A block design was used in all of these four runs. The experimental conditions were timed by presenting the green-colored word “GO” (for 2 s) indicating the beginning of the task (lasting 28 s) or the red-colored word “STOP” (for 2 s) informing participants to stop the task (control condition, lasting 18 s). For the motor execution run, participants performed four blocks comprising execution of right hand finger tapping movements while watching a fixation cross (28 s) and resting the right hand (18 s, control condition). Moto rexecution was performed in order to compare the recruitment of brain motor areas between different tasks (motor execution and imagery). Imagery runs (RUN1, RUN2 and RUN3) consisted of eight blocks alternating kinesthetic MI (28 s) and visual imagery (control condition, 18 s). During MI participants were required to perform kinesthetic imagery of right hand finger tapping after the GO signal (**Figure 1B**). Three seconds after the beginning of the block, dynamic bar graphs were shown and participants of both groups performed the MI while watching these (NFB or random) graphs. Participants were encouraged to perform the finger tapping MI task (and preceding real movement) at a stable frequency, chosen by each participant during the training phase before scanning. In the control condition (signaled by “STOP”), participants performed visual imagery of a static scene while watching a fixation cross on a LCD screen. This visual imagery was chosen by each participant and should not include humans, animals or movements. The scene could be a static photograph of a landscape, for example. We chose visual scene imagery as control condition because there were concerns that participants might find it difficult to switch between MI and “uncontrolled” rest, which could otherwise be associated with mind wandering (Porro et al., 1996). Participants of both groups had to perform visual imagery of a scene while watching a fixation cross.

Self-evaluation of arousal was performed before and after each run, and MI vividness rating was obtained after each MI run (both via the intercom). Similarly, rating on the Stanford Sleepiness Scale (SSS; Hoddes et al., 1972) was obtained after each run, to assess the level of arousal (7 point scale; anchor points 1 = feeling active, vital, alert or wide awake, 7 = having dream-like thoughts). For self-evaluation of vividness during MI, the kinesthetic subscale of the KVIQ-10 was employed; participants rated the vividness of MI experienced during the task on a 5-point scale (anchor points: 5 = as intense as execution, 1 = no sensation). These behavioral measures were collected to ensure that both groups would have similar levels of arousal and vividness of MI, and to allow valid group comparisons.

### MRI Data Acquisition

Functional images were acquired with a 3T Achieva scanner (Philips Medical Systems, the Netherlands) using an eight-channel SENSE head coil and a single-shot T2^∗^-weighted echoplanar imaging (EPI) sequence (*TR* = 2000 ms, *TE* = 30 ms, matrix 64 × 64, FOV 240 mm × 240 mm × 110 mm, flip angle = 90°, voxel size 3.75 mm × 3.75 mm, slice thickness = 5 mm, no gap, 22 slices). One hundred volumes were acquired in the motor execution run and 200 volumes in each imagery run. Before each run, five dummy volumes were collected for T1 equilibration purposes. A SENSE factor of 2 and “dynamic stabilization” were additionally employed (Foerster et al., 2005). Reference anatomical images were acquired using a T1-weighted three-dimensional magnetization-prepared, rapidly acquired gradient echo (MP-RAGE) sequence (TR/TE = 7.2/3.4 s, matrix/FOV 240/240 mm, flip angle = 8°, 1 mm isotropic voxel size, 170 sagittal slices). Head motion was restricted with foam padding and straps over the forehead and under the chin. The total MRI acquisition lasted about 34 min.

### Region of Interest (ROI) Selection for NFB

The left PMC was chosen as an ROI to be upmodulated during neurofeedback training due to its critical role in motor rehabilitation after brain lesions associated to motor impairment (Liu and Rouiller, 1999; Miyai et al., 1999; Fridman et al., 2004; Dancause et al., 2005). We defined the ROI based on a template HMAT from a meta-analysis (Mayka et al., 2006) in which authors used the activation likelihood estimation method based on a pool of 126 articles to estimate a 3-D anatomic boundary of PMC. The same ROI was used for all NFB group participants.

### Continuous Online Neurofeedback Data Analysis

During MI (“GO” blocks), participants received either real NFB or random visual stimuli. Continuous online neurofeedback data was performed using FRIEND (Functional Real-time Interactive Endogenous Neuromodulation and Decoding) toolbox^1^ (Sato et al., 2013; Basilio et al., 2015), which is a home-built toolbox for fMRI NFB. First, a linear registration between the EPI image (RFI) and a MNI template brain was obtained. Then, we calculated the inverse matrix of this transformation and applied it to the MNI ROI mask. For real-time feedback calculation, we used the motion corrected, gaussian smoothed EPI volumes (FWHM = 5 mm). For the NFB group participants, NFB information was calculated according to the formula:

(1) $$N\,F\,B\left(t\right)=\frac{B\,O\,L\,D\left(t\right)-B\,O\,L\,D\,\left(t_{s\,t\,o\,p}\right)}{B\,O\,L\,D\,\left(t_{s\,t\,o\,p}\right)}\,$$

where NFB(t) is the neurofeedback value at time *t*, BOLD(t) the average BOLD signal across voxels in the ROI at time *t* and BOLD(t_stop_) the average BOLD signal across voxels in the ROI and all time points of the preceding STOP condition block, rescaled to the interval 0–100% and fed back after each TR (2 s) only during the MI (GO) conditions. After each TR there was a 3 s delay related to the real-time processing. Graphs presented to CTL group participants were set up to provide randomly subtle level variations, visually equivalent to the real NFB.

### Oﬄine fMRI Data Analysis

Statistical Parametric Mapping (SPM8^2^) implemented in Matlab R2009b (The Mathworks INC^3^) was used for image analysis (Friston, 1995; Worsley and Friston, 1995). Functional datasets were pre-processed by realigning all volumes of each subject to the mean image generated for each run and realigning all four runs to each other, and by applying slice time correction. Functional images were co-registered and normalized to the standard MNI (Montreal Neurological Institute) EPI template, using 12-parameter affine normalization. The voxel dimensions of each normalized functional scan were kept in the original resolution of 3.75 mm × 3.75 mm × 5 mm. Functional images were also spatially smoothed using a 6 mm full-width half-maximum (FWHM) Gaussian kernel. Unwanted low frequencies in the fMRI time series were removed with high-pass filtering (128 s) and cubic detrending (Macey et al., 2004). Participants’ head movement analysis revealed that there was no movement greater than 4 mm.

In the first level analysis, pre-processed images of all four runs of each participant were analyzed with a General Linear Model comprising 2 predictors: (1) control condition (i.e., rest in motor execution runs and visual imagery of a scene in the MI runs, STOP); (2) experimental condition (i.e., execution of finger tapping in the motor execution run and kinesthetic MI of finger tapping in the MI runs, GO). Predictors were modeled as a boxcar function with a length of 18 s for (1) and 28 s for (2), convolved with the canonical hemodynamic response function (Zarahn et al., 1997). In the first level analyses, categorical contrasts were generated for GO vs. STOP (i.e., motor vs. visual imagery or motor execution vs. rest) for each run. We refer to the GO vs. STOP contrast of the last MI run as “RUN3” throughout the manuscript. In addition, for the MI runs the interaction contrast (GO RUN3 vs. STOP RUN3) vs. (GO RUN1 vs. STOP RUN1), is referred to as RUN3 vs. RUN1 for the sake of simplicity. The resulting contrast images (GO vs. STOP for motor execution, RUN3, and the interaction contrast RUN3 vs. RUN1) of each subject were submitted to a second level analyses using one sample *t*-tests for each group separately. For comparison between groups, (NFB vs. CTL), two sample *t*-tests were applied in the second level to (1) contrast images of RUN3 vs. RUN1, and (2) contrast images for RUN3. For this whole-brain analyses, significance was initially determined using a voxel-level threshold of *p* < 0.005 (uncorrected for multiple comparisons) and a minimum cluster size of five voxels, hereby increasing sensitivity for expected small effects. Activations were described using the Anatomy toolbox for SPM8^4^. ROI analyses (see **Figure 3B**) were performed by using anatomically defined *a priori* ROIs selected from anatomic atlases: left and right PMC, left M1, and SMA (template HMAT, Mayka et al., 2006); left and right BG (Putamen, Caudate, WFU PickAtlas^5^) and right cerebellum (Lobule VI; Anatomy toolbox).

### Accelerometer Measurements

During the fMRI sequences, hand movement was monitored with an acceleration sensor (Brain Products 3D Acceleration Sensor MR; sensitivity: 420 mV/g; Supply voltage: ±5 V DC), which was attached to the right thumb. Root mean square (RMS) values of the *x,y,z*-acceleration values were calculated for each condition (MI, visual imagery) in each run and used to determine the ratio between motor vs. visual imagery.

### Statistical Analysis of Behavioral Data

Behavioral data (arousal scores, vividness scores, accelerometer) were analyzed with SPSS 20.0 (IBM Corporation, New York) using repeated measure analyses of variance (ANOVA) with the within subject factor run (RUN3 and RUN1) and between subject factor group (NFB and CTL). The Greenhouse Geiser correction was applied for correcting *p*-values in cases in which sphericity was violated. Comparisons of groups for mental chronometry scores and scores obtained from the KVIQ-10 were done using two sample unpaired *t*-test (two-tailed).

## Results

### Behavioral Data

Because NFB participants might have considered their MI task as being more challenging than the CTL group participants, we compared arousal and vividness of MI ratings between groups. Using repeated measure ANOVAs with the within-subject factor RUN (1,3) and the between-factor Group (NFB and CTL), we found no significant differences in arousal (measured with the SSS) between the two groups (no main effect group and no interaction group × run, *F*s < 1). Sleepiness increased over the course of the runs in both groups [main effect RUN, *F*(1,26) = 7.8, *p* = 0.01; **Table 1B**; note that all values fell below three indicating that participants were awake]. The CTL group reported slightly more subjective vividness of MI following MI runs, [*F*(1,26) = 4.1, *p* = 0.05; **Table 1B**]. Neither main effects for RUN nor interactions of RUN x group were found for vividness (*F*s < 1). Furthermore, no statistical differences were observed for mental chronometry scores [*t*(25) *<*1] and general MI ability (KVIQ-10) between groups [visual MI: *t*(26) = 1, *p* = 0.32; kinesthetic MI: *t*(26) < 1; **Table 1A**]. These results suggest that there were no differences in the ability to perform MI between the NFB and CTL groups.

### Accelerometer Measures

In order to evaluate whether participants might have inadvertently moved when performing the imagery tasks during fMRI scanning, we monitored right thumb movement using an accelerometer. Due to availability of the equipment, movements of only 19 out of 28 participants (7 NFB, 12 CTL) could be monitored with this device. Still, this data allowed us to test whether there were differences between groups and runs over time (**Table 1B**). A repeated-measures analysis with the within-subject factor RUN (RUN1 and RUN3) and the between-subject factor Group (NFB and CTL) revealed that there were neither a main effect for Group [*F*(1,17) = 2.4, *p* = 0.14] or RUN [*F*(1,17) = 2.7, *p* = 0.12] nor any interaction between these factors [*F*(1,17) = 3.7, *p* = 0.07] on the movement measurements (ratio for movement during MI vs. visual imagery). In addition to the accelerometer quantitative measures, visual inspection of participant’s hand was performed during the MI runs in all participants. No hand movement was detected during the MI task.

### fMRI Data

#### RUN3 vs. RUN1 Comparison

**FIGURE 2:**
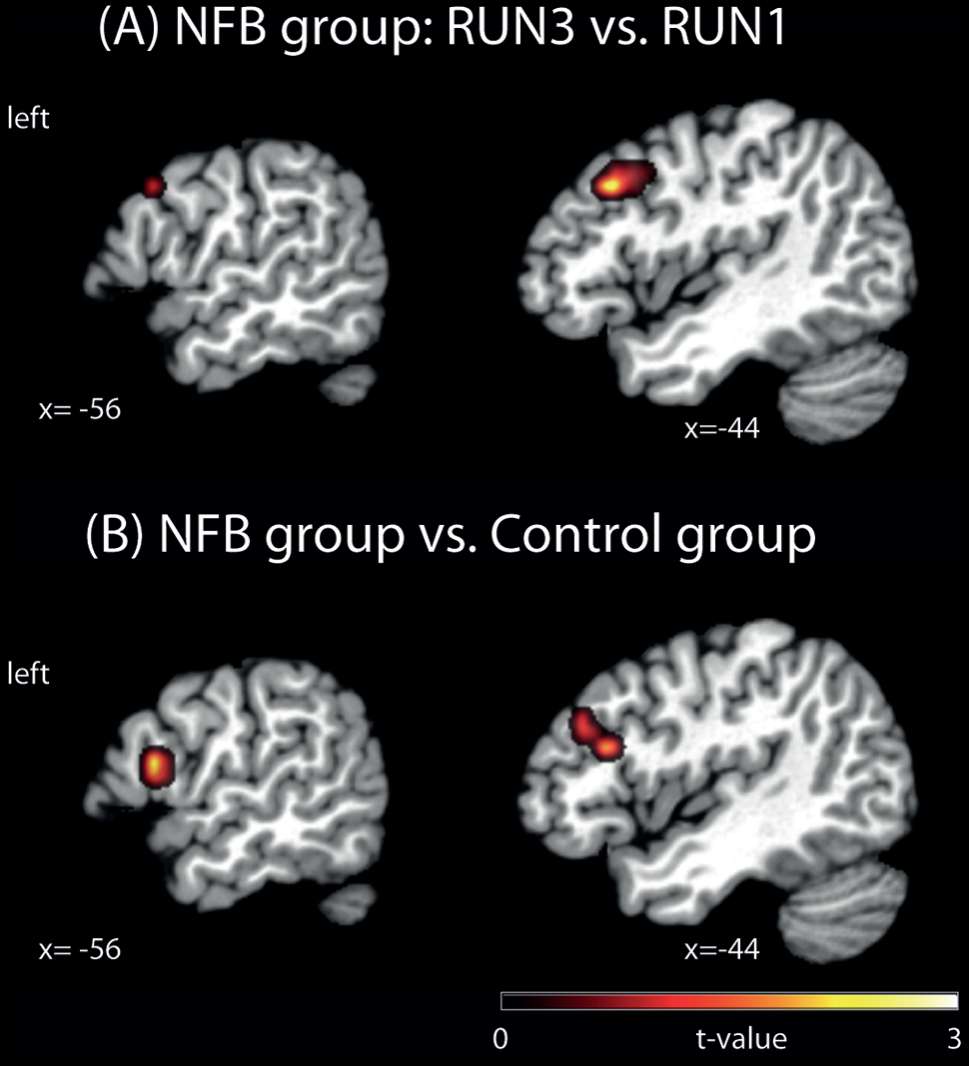
**Brain activation clusters for **(A)** the NFB group comparing RUN3 vs. RUN1; **(B)** NFB vs. CTL group**. The depicted activations in **(A)** and **(B)** are significant at a threshold of *p* < 0.005, uncorrected with a minimum cluster size of *k* = 5. The coordinates are given according to MNI space and activations are plotted on the MNI standard brain. MNI, Montreal Neurological Institute.

To investigate if healthy volunteers are able to increase the activation of left PMC during NFB training, we directly compared brain activity during MI vs. visual imagery of RUN3 (last run) with the brain activity of the same contrast of RUN1 (first run). The NFB group (**Figure 2A**, **Table 2A**) showed increased activation of the NFB-targeted region (left PMC) compared to RUN3 vs. RUN1, indicating that this brain region in the NFB group was more activated at the end of the NFB training than in the beginning. In addition, clusters on the superior frontal gyrus, middle frontal gyrus, and the hippocampus showed increased activation at the end of the NFB training (**Table 2A**). Brain activation in the CTL group showed no difference between RUN3 and RUN1 in the PMC (**Table 2B**).

**Table 2:** Comparison of brain activations in the last (RUN3) vs. first (RUN1) runs in the NFB group and in the CTL group^∗^.

Anatomical region	Hemisphere	Cluster size (mm^3^)	MNI coordinates			*Z*-score
			*x*	*y*	*z*	
**(A) NFB RUN3 vs. RUN1**						
Superior frontal gyrus	R	352	12	53	45	3.63
Premotor cortex/BA44	L	844	-48	23	35	3.50
Hippocampus	L	563	-29	-14	-10	3.06
Middle frontal gyrus	L	352	-22	19	50	2.94
**(B) CTL RUN3 vs. RUN1**						
Cerebellum	R	352	27	-30	-25	3.27

*^∗^Values represent MNI coordinates for significant activation maxima of clusters in the one sample *t*-test random effects analyses (*p* < 0.005, uncorrected with a minimum cluster size of *k* = 5). BA, Brodmann area*.

#### NFB vs. CTL Group Comparison

In order to test whether the increased PMC activation observed in NFB group was significantly different from any (non-significant) changes in the CTL group, we contrasted RUN3 vs. RUN1 between groups. At the end of the neurofeedback training, the activation in a cluster located in the PMC (BA44) and the left inferior frontal gyrus (pars triangularis, BA45) was higher in the NFB than CTL group (**Figure 2B**, **Table 3A**), when comparing to the beginning. Lowering the threshold to *p* < 0.01 uncorrected revealed that the cluster in the left PMC was partially overlapping with the cluster identified for the NFB group (RUN3 vs. RUN1) alone (see Supplementary Figure S1). By taking the results obtained within the CTL group as the reference and comparing with the NFB group, no remaining activation in the PMC was observed (**Table 3B**).

**Table 3:** Comparison of brain activations for the last (RUN3) vs. first (RUN1) runs contrasting the NFB and the CTL groups^∗^.

Anatomical region	Hemisphere	Cluster size (mm^3^)	MNI coordinates			*Z*-score
			*x*	*y*	*z*	
**(A) NFB vs. CTL**						
PMC/BA44	L	422	-59	16	10	3.12
IFG/BA45	L	562	-44	27	20	3.08
**(B) CTL vs. NFB**						
Parietal Operculum	L	422	50	-3	10	3.25

*^∗^Values represent MNI coordinates for significant activation maxima of clusters in the two sample *t*-test random effects analyses (*p* < 0.005, uncorrected with a minimum cluster size of *k* = 5). BA, Brodmann area; PMC, premotor cortex; IFG, inferior frontal gyrus*.

#### Comparison of Brain Motor Areas During Motor Imagery between Groups

**FIGURE 3:**
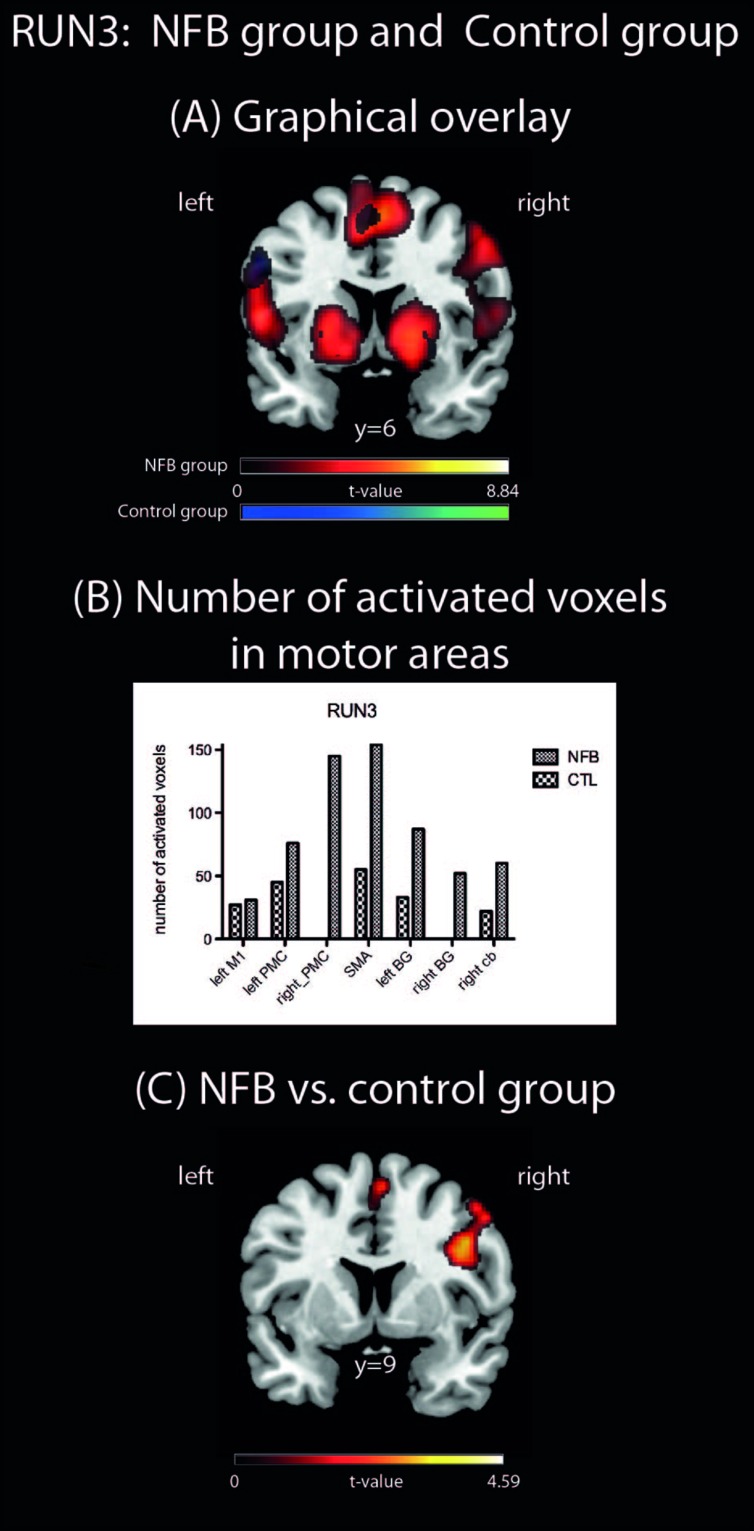
**Brain activations during RUN3. (A)** A Graphical overlay of activation maps of the last imagery run (RUN3, contrast: motor vs. visual imagery) for the NFB (red scale) and the CTL (blue scale) groups; **(B)** number of activated voxels in motor brain areas during RUN3 using anatomically pre defined regions of interest (left M1, right PMC, SMA, left and right basal ganglia, right cerebellum, including the left PMC). Cb, cerebellum; **(C)** comparison of brain activation during RUN3 contrasting NFB vs. CTL group. The depicted activations in **(A)** and **(C)** are significant at a threshold of *p* < 0.005, uncorrected with a minimum cluster size of *k* = 5. The coordinates are given according to MNI space and activations are plotted on the MNI standard brain.

In order to assess how the MI-associated motor regions might have been modulated by NFB training, we compared the pattern of brain activation during the last run (RUN3) of the MI task between the groups (NFB and CTL, **Figure 3**). The graphical overlay suggested that the activation of motor-related brain areas during the last run of MI (RUN3) in NFB group was noticeably more extensive than those observed in CTL group during the same run (**Figure 3A**; **Table 4**). The NFB group showed higher number of activated voxels in different predefined motor regions [**Figure 3B**; ROIs: left M1, SMA, bilateral PMC, bilateral BG, and right anterior cerebellum; sum over all voxels in ROIs: NFB mean ± SD: 86.43 ± 17.64; CTL mean ± SD: 26.00 ± 7.898; unpaired *t*-test, *t*(12) = *p* < 0.01]. Indeed, direct comparison between groups (**Figure 3C**; **Table 5**) showed that the NFB group had significantly higher activation in right (ipsilateral) PMC and SMA during the last MI run compared to CTL group. The reverse comparison (CTL vs. NFB group) showed no significant activation in motor areas, even at very lenient thresholds (**Table 5**).

**Table 4:** Brain activation during RUN3 in the NFB and CTL groups^∗^.

Anatomical region	Hemisphere	Cluster size (mm^3^)	MNI coordinates			*Z*-score
			*x*	*y*	*z*	
**(A) NFB group**						
SMA	R/L	12654	1	4	55	5.96
Basal Ganglia/Thalamus	R/L	27417	-26	4	0	4.95
Cerebellum (lobule VI)	R	5624	23	-63	-25	4.82
IPC	R	11389	38	-48	45	4.72
PMC (BA44/6)	L	6538	-56	4	10	4.61
PMC(BA44/6)	R	22918	53	4	45	4.56
MFG	R	2390	38	42	20	4.07
IPC	L	3445	-63	-41	35	3.90
PMC (BA6)	L	4218	-44	-7	45	3.59
IPC/BA2	L	2882	-40	-44	50	3.56
Left MTG	L	352	-48	-56	0	3.07
**(B) CTL group**						
SMA	R/L	3867	-7	-3	55	4.68
Cerebellum (lobule VI)	R	1547	27	-60	-30	4.32
PMC (BA6)	L	4148	-26	-11	55	4.24
PMC (BA6/44)	L	1547	-56	4	35	4.15
Basal Ganglia/Thalamus	R/L	3585	-26	0	-5	3.86
Precuneus/WM	R	2109	16	-37	15	3.80
Precuneus/WM	L	773	-29	-56	5	3.39
IPC/Operculum	L	703	-59	-30	20	3.31
IPC	L	844	-37	-41	50	3.27
Basal Ganglia	R	562	23	4	5	2.95

*^∗^Values represent MNI coordinates for significant activation maxima of clusters in the one sample *t*-test random effects analyses (*p* < 0.005, uncorrected with a minimum cluster size of k = 5). BA, Brodmann area; PMC, premotor cortex; IPC, inferior parietal cortex; MFG, middle frontal gyrus; MTG, middle temporal gyrus; WM, white matter*.

**Table 5:** Comparison of brain activations for the last (RUN3) run of motor imagery between NFB and CTL groups^∗^.

Anatomical region	Hemisphere	Cluster size (mm^3^)	MNI coordinates			*Z*-score
			*x*	*y*	*z*	
**(A) NFB RUN3 vs. CTL RUN3**						
SMA	R/L	3515	4	12	60	3.90
Superior occipital gyrus	R	2461	23	-71	40	3.73
IPC	R	633	61	-41	35	3.69
PMC	R	4218	53	4	45	3.66
ITG	R	773	53	-48	-15	3.53
MFG	R	1476	38	53	25	3.40
IPC	R	562	38	-52	45	3.24
Precentral gyrus	R	352	34	-3	45	3.18
IFG	R	984	27	27	-15	3.11
Intraparietal sulcus	L	352	-29	-56	40	2.95
**(B) CTL RUN3 vs. NFB RUN3**						
Parietal operculum	R	844	46	-7	15	3.34
IFG	L	422	-33	42	-15	3.00

*^∗^Values represent MNI coordinates for significant activation maxima of clusters in the one sample *t*-test random effects analyses (*p* < 0.005, uncorrected with a minimum cluster size of *k* = 5). BA, Brodmann area; IPC, inferior parietal cortex; IFG, inferior frontal gyrus; MFG, middle frontal gyrus; IFG, inferior frontal gyrus*.

#### Similarities Between Activated Regions During Motor Execution and Motor Imagery

**FIGURE 4:**
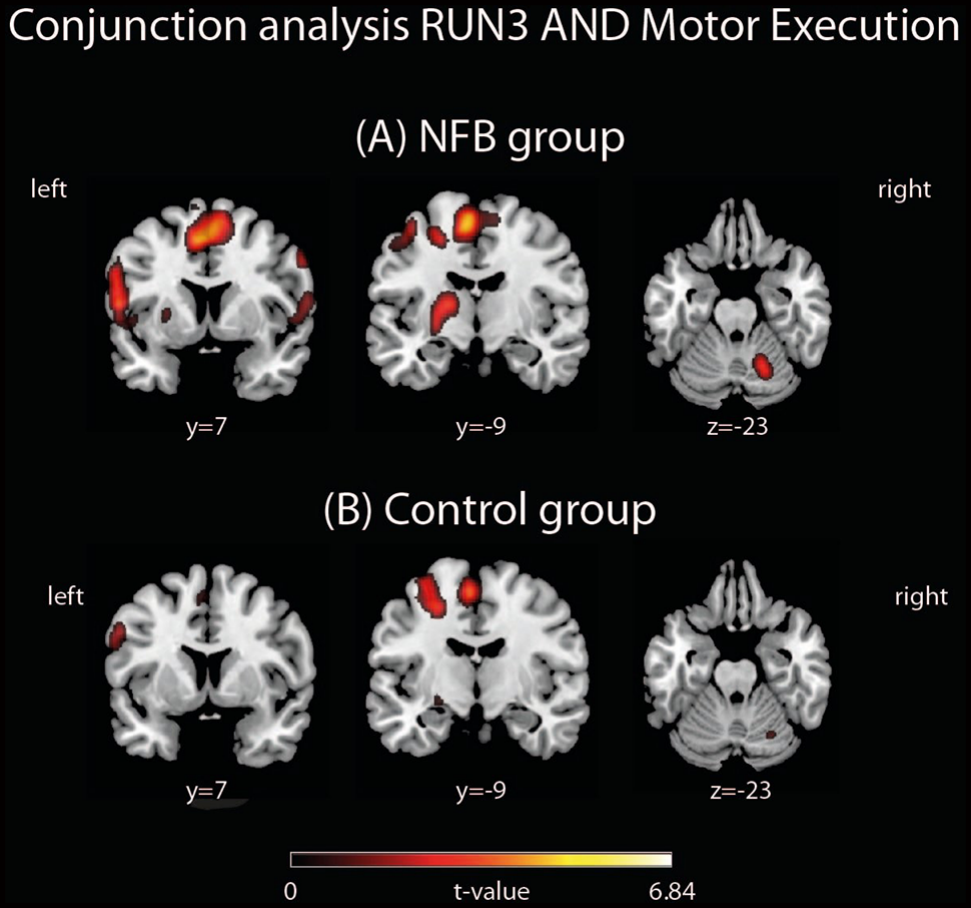
**Conjunction analyses between RUN3 and Motor Execution in the **(A)** NFB and **(B)** CTL groups**. The depicted activations in **(A)** and **(B)** are significant at a threshold of *p* < 0.005, uncorrected with a minimum cluster size of *k* = 5. The coordinates are given according to MNI space and activations are plotted on the MNI standard brain.

We examined a possible effect of NFB training on how similar the MI network in RUN3 became to that observed during the motor execution task. In other words, we asked if NFB training increased the overlap between activity in response to real and imagined finger tapping. To this aim, conjunction analyses were performed separately for the NFB and CTL group in order to identify brain areas activated in both conditions: MI (RUN3) and motor execution (**Figure 4**). These analyses revealed that the number of commonly activated voxels during both motor execution and MI was three times greater in NFB than in CTL group (whole brain analysis; 405 activated voxels in NFB group and 135 in CTL group; **Table 6**).

**Table 6:** Common brain regions activated during both motor execution and motor imagery during the last run (RUN3) assessed by conjunction analysis.

Anatomical region	Hemisphere	Cluster size (mm^3^)	MNI coordinates			*Z*-score
			*x*	*y*	*z*	
**NFB group**						
SMA	R/L	10686	-7	-3	55	5.13
PMC (BA6/44)	L	4921	-56	4	10	4.32
Cerebellum (lobule VI)	R	2039	23	-60	-30	3.88
Basal Ganglia/Thalamus	L	5062	-26	-3	-5	3.72
PMC (BA6)	R	492	57	4	40	3.58
IPC	R	2250	50	-33	40	3.43
MFG/BA6	L	1055	-26	-7	50	3.39
PMC (BA44/Rolandic Operculum)	R	1547	61	8	15	3.38
Amygdala	R	422	20	0	-10	3.36
**CTL group**						
SMA	R/L	3656	-3	-3	60	5.19
PMC (BA6)	L	2601	-29	-11	60	3.87
PMC (BA6/44)	L	1125	-56	4	35	3.86
Cerebellum (lobule VI)	R	1265	27	-67	-30	3.54
IPC	L	422	-59	-30	20	3.14
Amygdala	L	422	-26	-7	-10	3.03

*(MOTOR ∩ RUN3)^∗^^∗^Values represent MNI coordinates for significant activation maxima of clusters in the one sample *t*-test random effects analyses (*p* < 0.005, uncorrected with a minimum cluster size of *k* = 5). BA, Brodmann area; IPC, inferior parietal cortex; STG, superior temporal gyrus*.

## Discussion

Here we investigated whether healthy human volunteers are able to increase activation of the left PMC during a right-hand finger tapping MI task while receiving NFB information, in a controlled study. A NFB experimental group received ongoing feedback (displayed in bar graphs on a LCD screen) representing their activity in the left PMC. A CTL group observed random graphs displayed on the LCD similarly to the NFB group, but was informed that these graphs were random images in the LCD screen. We compared brain activations of the NFB group during MI of the last (third) run with those measured in the first run and found increased activation in the left PMC. The CTL group did not show increase in activation in the PMC. The comparison between brain activations of the NFB and the CTL group showed a resultant activation slightly rostral to the PMC, in BA 44. Both activations (within NFB group and between groups) were localized partially outside the ROI used for the NFB. Single run results (**Figure 3**) showed that the peak activation was located within this ROI. Further analyses were performed in order to investigate whether the modulation of left PMC was accompanied by activation in the other components of the motor control brain areas. During RUN3 the NFB group showed strong activation in left and right PMC, left and right BG, left M1, right anterior cerebellum and bilateral SMA. These results suggest that the local modulation of left PMC was followed by additional recruitment of both ispi- and contralateral motor related areas. Indeed, contrasting RUN3 between groups showed that this effect seemed to be more substantial over SMA and right (ipsilateral) PMC. We also investigated how similar the MI network became to that observed during motor execution. Conjunction analyses between RUN3 and motor execution revealed that the NFB group had a much higher overlap among the brain regions activated during motor execution and MI, as compared to the CTL group.

Our results support the hypothesis that the NFB group would be able to increase the activation of left PMC during NFB training (**Figure 2A**, **Table 2**). On the other hand, the CTL group did not show significant increase in left PMC activation across MI runs, ruling out the possibility that the effects observed in the NFB simply reflected an effect of MI training over the course of time. In line with previous studies employing fMRI (deCharms et al., 2004; Yoo et al., 2008; Chiew et al., 2012), we showed that MI task during NFB training is an effective tool to provide up-regulation of motor-related brain areas. In a recent exploratory study, Sitaram et al. (2011) implemented a NFB paradigm focusing on modulation of brain activity in the ventral premotor area in two stroke patients and four healthy volunteers. Despite the small sample, these investigators were able to show an increase in ROI activation after days of NFB practice. To our knowledge our study presents the first controlled study that demonstrates the increment of left PMC activation over NFB training sessions. The importance of PMC recruitment has been emphasized by recent reports on motor rehabilitation. Studies employing transcranial magnetic stimulation have shown that the PMC seems to play a critical role in motor recovery after stroke followed by motor impairment (Johansen-Berg et al., 2002; Fridman et al., 2004). Furthermore, both anatomical and functional plasticity that follow brain lesions associated with motor impairments support these findings (Liu and Rouiller, 1999; Miyai et al., 1999; Dancause et al., 2005). Interestingly, our results showed additional recruitment of distant brain areas involved in motor control, such as the SMA, ipsilateral PMC, BG, and cerebellum, during NFB training. These results might contribute for future application in stroke patients, for example, since evidences suggest that activation in spared motor brain areas, especially in more impaired stroke patients, posivitly correlates with motor performance (for a review, see Ward, 2004). In addition, the brain activation comparison between groups in our study is in line with a near-infrared spectroscopy-based NFB study, that showed a rostral-oriented enlargement of activation through training (Mihara et al., 2012). Although with similarities among experimental designs, we took advantage from the high anatomical resolution of the MRI approach, alowing the investigation of cortical and subcortical brain responses to NFB training.

Despite the CTL group revealed slight increase in vividness during MI at the end of runs (*p* = 0.05), this effect did not result in stronger activation in motor areas. One possible explanation for this finding is the fact that the NFB group received feedback about their brain activity, which was adapted to their level of activation and thus always allowed for training and improvement. On the other hand, individuals in the CTL group counted on their own subjective perception of their efforts alone. Therefore, individuals in the NFB group might have evaluated their own vividness more critically and as being less intense. Importantly, there was no difference in self-reported sleepiness between groups and no decrease in MI vividness over the course of the experiment.

No statistical differences were found between groups in their individual ability to perform MI, as evidenced by measures of the kinesthetic and visual imagery questionnaire and the mental chronometry task. This supports the assumption that both NFB and CTL groups would be equally capable to perform MI during fMRI scanning. A previous study has shown that MI during NFB training is feasible without hand muscle contractions in healthy volunteers (Berman et al., 2012). In line with this, the measures of thumb movements during both visual and MI during fMRI scanning did not show substantial movements, and there were no significant differences between groups (as quatified by the acelerometer). Similar to previously fMRI NFB studies (deCharms et al., 2004; Yoo et al., 2008; Megumi et al., 2015), we have not directly recorded muscle activity measures, unfortunatly. Although unlikely, undetected spontaneous (micro) movement patterns might have had an impact on our results. As such we cannot definitively rule out possible brain responses to such slight muscle contraction that do not lead to movement.

Some further methodogical caveats need to be considered. In contrast to most studies in the field (deCharms et al., 2004; Yoo et al., 2008; Hamilton et al., 2011; Chiew et al., 2012; Hui et al., 2014), participants of the CTL group in our study were aware of the random nature of the graphs they were exposed to during the experiment. We purposefully chose this design because we hypothesized that providing false feedback information about left PMC activity could increase frustration and thus reduce the recruitment of motor brain areas that would be naturally elicited by MI alone. Indeed, previous studies suggested that the neurophysiological effect of the sham group throughout NFB training might overestimate the differences between groups (deCharms et al., 2005; Caria et al., 2010; Megumi et al., 2015) or elicit placebo effects (Sulzer et al., 2013). We believed that our instructions would not influence the performance of MI in the CTL group, providing an adequate comparison for estimating the effect of real NFB training. On the other hand, the nature of the tasks performed by each group was obviously different. Whereas the CTL group was engaged in merely imagining finger tapping movements with their right hand, the NFB group had to perform the same task in addition to watch the bar graph’s behavior and evaluate strategies that would allow an increase in BOLD signal. Thus, the NFB group performed a task with a higher cognitive demand, which could by itself lead to recruitment of brain regions involved in executive processes, such as anterior cingulate cortex (Cavanagh et al., 2012). Our results indicate that specific motor control areas, previously associated with MI (Munzert et al., 2009), but not regions associated with general attention, effort and executive processes, were recruited. This selective increase may reflect a strengthening of the motor control network as a whole, instead of a general increase in cognitive demands. Since our control stimuli could introduce some confunding factors, such as sleepiness, we collected behavioral measurements regard participants’ sleepiness and MI vividness before and after each MI run. Because no diferences among groups were present, our results suggest that these possible confounding factors did not have a substantial impact on the results. It remains an open question, however, how the approach employed herein for the CLT group compares with the more traditional sham neurofeedback one, and whether the use of different modalities of stimuli (visual, tactile, etc) for feedback would lead to different results in neurofeedback training (for review, see Sulzer et al., 2013). More studies are thus needed in order to optimize neurofeedback protocols and to evaluate the most appropriate control tasks. In the present study we adopted visual imagery as a control condition in order to avoid mind wandering or eliciting unwanted cognitive processes during an “uncontrolled” resting task. A further reason for choosing an active control condition such as visual imagery was that there were some concerns that switching between MI and “uncontrolled” stop/resting condition might be difficult for some participants (Porro et al., 1996).

A number of studies have employed a control ROI (normally a region supposedly not involved in the task of interest) in order to control for noise and other non-specific effects (deCharms et al., 2004, 2005; Caria et al., 2007). We chose not to use a control ROI in the present study because such global or non-physiological effects often show linear or non-linear trends that vary substantially across regions, such that local signal changes may not reflect global effects. Moreover, a control region might also contain signal changes that are of interest, thereby reducing signal-to-noise in the target ROI. In our piloting, unpublished results, we did not observe advantages when subtracting control region signals from the target ROI signal. We believe that future studies are necessary to provide solid evidence for the benefit of using signals from control brain regions as a way to improve signal-to-noise in neurofeedback experiments.

Some limitations of our study need to the considered. Our reported main effects were not very robust, and are reported at uncorrected statistical significance levels (though large cluster sizes were found in many regions, we did not attempt to use cluster-based or threshold-free corrections and chose to adopt the more conventional SPM reporting). Several explanations and consideration might account for this. First, we compared the last neurofeedback training run to the first one, instead of employing a low-level, non-neurofeedback baseline condition. This might have led to an underestimation of the neurofeedback-induced BOLD increases. Second, the neurofeedback training was relatively short (only one session, lasting less than 1 h). Additional training sessions probably would have further increased the effects. Third, our sample size of *N* = 14 in each group is limited and might have contributed to the limited power to detect more significant effects. Of note, previous studies on MI-related activations generally show smaller effects compared to motor execution (Stephan et al., 1995; Lotze et al., 1999; Gerardin et al., 2000; Hanakawa et al., 2003). This may be the reason why some studies using MI have reported results at uncorrected levels (Gerardin et al., 2000; Hanakawa et al., 2003). Taken together, we believe that our results are strongly supported by previous studies and corroborate our *a priori* hypothesis, despite the caveats discussed above.

Our study employed a freely available rt-fMRI software (FRIEND^6^; Sato et al., 2013; Basilio et al., 2015). This may contribute for expansion of the NFB fMRI use and allow a better comparability across studies, which is fundamental for future applications in clinical research. Because MI has an important effect on motor rehabilitation after stroke (Sharma et al., 2006), future studies should investigate whether MI tasks reinforced by fMRI NFB provides further help for stroke motor recovery.

## Conclusion

Our results suggest that healthy individuals are able to increase the activation of a crucial brain region involved in motor control, the left PMC, via fMRI NFB training. Furthermore, this procedure induced recruitment of wider brain motor regions during the MI task. These results in healthy participants may represent an important step toward clinical applications of fMRI neurofeedback in rehabilitation after stroke or other motor disabilities.

## Author Contributions

TFM, RB, ECR, FT-M designed the research; TFM, ECR, AE, SH, RL, JM, FT-M performed the research; TFM, AE, SH, ECR, RL, JM, FT-M wrote the paper.

## Conflict of Interest Statement

The authors declare that the research was conducted in the absence of any commercial or financial relationships that could be construed as a potential conflict of interest.

## Footnotes

1 http://fsl.fmrib.ox.ac.uk/fsl/fslwiki/OtherSoftware

2 www.fil.ion.ucl.ac.uk/spm/software/spm8

3 http://www.mathworks.com

4 www.fz-juelich.de/ime/spm_anatomy_toolbox

5 http://fmri.wfubmc.edu/software/PickAtlas

6 https://github.com/InstitutoDOr/FriendENGINE
