# Supplementary material for: Enhancing Motor Network Activity Using Real-Time Functional MRI Neurofeedback of Left Premotor Cortex
Source: Front Behav Neurosci. 2015 Dec 24;9:341. doi: 10.3389/fnbeh.2015.00341 (PMC4689787; doi:10.3389/fnbeh.2015.00341)
Supplement: Supplementary file 1 [file Supplementary_Figure_S1.PDF]

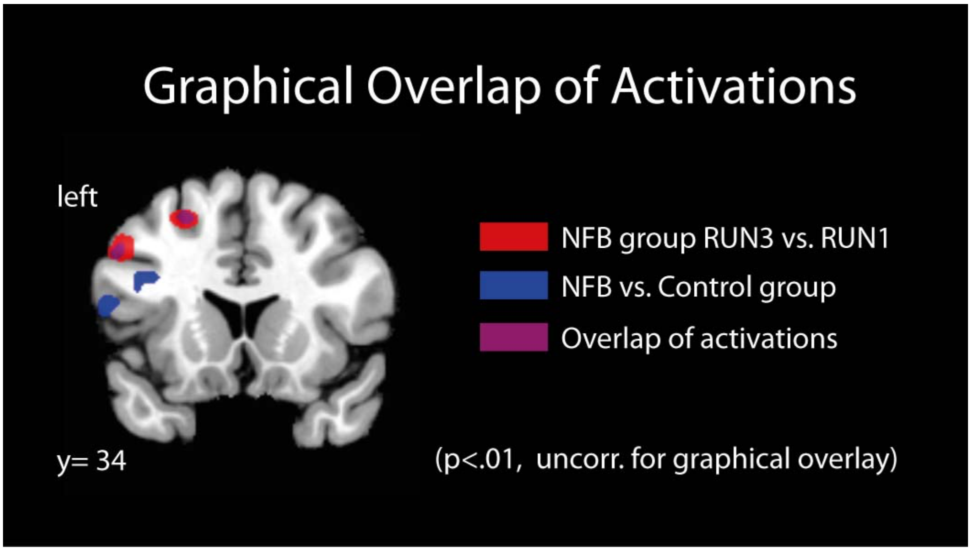

Figure S1: Graphical overlap of the clusters identified in the NFB comparing RUN3 vs. RUN1 and in the comparison of the NFB vs. CTL groups. For explorative reasons the depicted activations were displayed at a significance threshold of $p < 0.01$, uncorrected. Results of the contrast comparing RUN3 vs. RUN1 in the NFB group are shown in red, results of the comparison of the NFB vs. CTL group are displayed in blue and their spatial overlap is shown in violet. The coordinates are given according to MNI space and activations are plotted on the MNI standard brain. MNI: Montreal Neurological Institute.
